# P004. Evidence based psychological treatments in pain management: a review of controlled and randomized trials about chronic headache, neuropathic pain and fibromyalgia

**DOI:** 10.1186/1129-2377-16-S1-A151

**Published:** 2015-09-28

**Authors:** Amerigo Costa, Francesco Melina, Alessandra Sansalone, Rosario Iannacchero

**Affiliations:** Centre for Headache and Adaptive Disorders, Unit of Neurology, Department of Neuroscience and Sense Organs, Azienda Ospedaliera “Pugliese-Ciaccio”, Catanzaro, Italy; Psychiatric Service for Diagnosis and Treatment, Department of Mental Health, Azienda Sanitaria Provinciale, Catanzaro, Italy

## Background

Pain therapy settings often offer psychological interventions complementary to biomedical treatments [1]. While a large amount of scientific literature exists, there is relatively little experimental level evidence about the efficacy of psychotherapy in chronic pain. We reviewed randomized and controlled studies about the efficacy of psychotherapy in neuropathic pain conditions (NP), fibromyalgia (FM) and chronic headache (CH).

## Materials and methods

In March 2015, we searched the Cochrane Central Register of Controlled Trials using the keywords “psychotherapy”, “chronic headache”, “neuropathic pain”, and “fibromyalgia”. We excluded non-randomized studies and identified works involving psychotherapeutic approaches showing evidence of efficacy. We performed descriptive statistics over quantitative and qualitative data.

## Results

**Figure 1:**
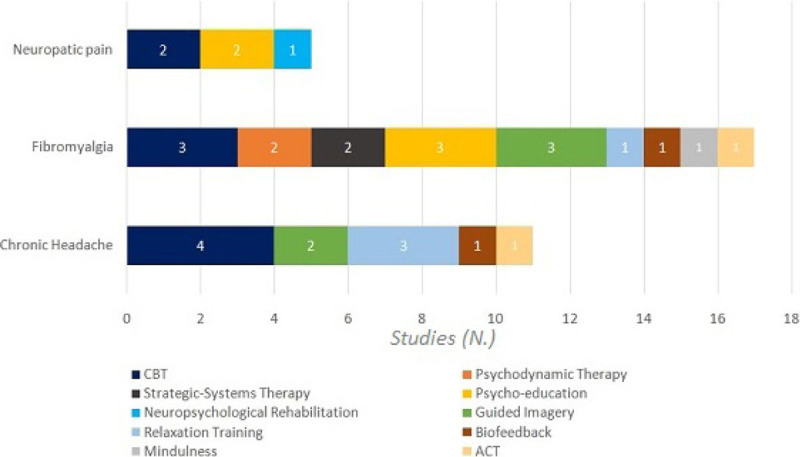
Controlled and randomized studies about psychological treatments for chronic headache, neuropathic pain and fibromyalgia.

Concerning neuropathic pain conditions, we found evidence for Cognitive-Behavioral Therapy (CBT) (2 studies), Psycho-education (PE) (2 studies) and Neuropsychological Rehabilitation (1 study). Regarding fibromyalgia, we found evidence for CBT (3 studies), PE (3 studies), Guided Imagery (GI) (3 studies), Strategic-Systems Therapy (Ericksonian) (2 studies), Brief Psychodynamic Therapy (1 study), Relaxation Training (RT) (1 study), Acceptance and Commitment Therapy (ACT) (1 study), Biofeedback (1 study), Mindfulness (1 study). We found evidence for CBT (4 studies), RT (3 studies), GI (2 studies), Biofeedback (1 study), ACT (1 study) in the treatment of chronic headache (Figure 1). It is noteworthy that CBT studies often involved informational group meetings; brief psychodynamic therapy was always carried out as a group intervention rather than individual sessions; strategic-systems therapy always involved hypnosis as elaborated in the Milton H. Erickson model of brief therapy.

## Conclusions

There is considerable terminological overlap between psychological approaches used in pain management. Many of the above-mentioned methods refer to similar practices under different names and therapists use them under different theoretical models. Our review supports the hypothesize that informative (psycho-education) and psycho-physiological interventions (biofeedback; relaxation training; guided imagery; mindfulness; ACT; neuropsychological rehabilitation) integrated with psychotherapy models (CBT; psychodynamic therapy; strategic-systems therapy) are useful in managing the considered forms of chronic pain.
